# Role of mesenchymal cells in the natural history of ovarian cancer: a review

**DOI:** 10.1186/s12967-014-0271-5

**Published:** 2014-10-11

**Authors:** Cyril Touboul, Fabien Vidal, Jennifer Pasquier, Raphael Lis, Arash Rafii

**Affiliations:** Department of Obstetrics and Gynecology, Hôpital Intercommunal de Créteil, Université Paris Est, UPEC-Paris XII, 12 avenue de Verdun, 94000 Créteil, France; UMR INSERM U965: Angiogenèse et Recherche translationnelle Hôpital Lariboisière, 49 bd de la chapelle, 75010 Paris, France; Stem Cell and Microenvironment Laboratory, Weill Cornell Medical College in Qatar, Education City, Qatar Foundation, Doha, Qatar; Department Genetic Medicine, Weill Cornell Medical College, Manhattan, NY USA; Department of Genetic Medicine and Obstetrics and Gynecology, Stem Cell and Microenvironment Laboratory, Weill Cornell Medical College in Qatar, Qatar-Foundation PO: 24144, Doha, Qatar

**Keywords:** Mesenchymal stem cells, Ovarian cancer, Crosstalk, Phenotypic modulation, Dissemination, Chemoresistance

## Abstract

**Background:**

Ovarian cancer is the deadliest gynaecologic malignancy. Despite progresses in chemotherapy and ultra-radical surgeries, this locally metastatic disease presents a high rate of local recurrence advocating for the role of a peritoneal niche. For several years, it was believed that tumor initiation, progression and metastasis were merely due to the changes in the neoplastic cell population and the adjacent non-neoplastic tissues were regarded as bystanders. The importance of the tumor microenvironment and its cellular component emerged from studies on the histopathological sequence of changes at the interface between putative tumor cells and the surrounding non-neoplastic tissues during carcinogenesis.

**Method:**

In this review we aimed to describe the pro-tumoral crosstalk between ovarian cancer and mesenchymal stem cells. A PubMed search was performed for articles published pertaining to mesenchymal stem cells and specific to ovarian cancer.

**Results:**

Mesenchymal stem cells participate to an elaborate crosstalk through direct and paracrine interaction with ovarian cancer cells. They play a role at different stages of the disease: survival and peritoneal infiltration at early stage, proliferation in distant sites, chemoresistance and recurrence at later stage.

**Conclusion:**

The dialogue between ovarian and mesenchymal stem cells induces the constitution of a pro-tumoral mesencrine niche. Understanding the dynamics of such interaction in a clinical setting might propose new therapeutic strategies.

## Introduction

Ovarian cancer is the most lethal gynecologic malignancy in developed countries, responsible for 5.8% of cancer related deaths [1]. The mainstay of treatment involves cytoreductive surgery associated with platinium and taxane-based chemotherapy [2]. Despite tremendous progresses in surgical practice and the broad range of chemotherapy or targeted therapy available, only low improvement has been achieved in survival outcomes these past 10 years [3-11]. Patients’ clinical course remains unpredictable, although most of them are optimally treated, with no residual disease after surgical debulking: 60% of women presenting with advanced-stage at baseline will recur within 5 years [12,13]. Therefore, 5-year overall survival remains low (around 45% including all stages) mainly due to peritoneal recurrences, suggesting the existence of occult sanctuaries where cancer cells are protected against treatment. Several authors have determined tumor autonomous parameters associated with treatment resistance [14,15]. Moreover, heterogeneity of ovarian cancers between and within subtypes has been illustrated by transcriptomic and genetic profiling [16]. Finally copy number variation analysis has revealed significant differences between matched primary ovarian tumor and peritoneal metastases [17]. Beyond these cell autonomous features, tumor environment may also contribute to the development of clinical relapse.

Tissues are comprised of different cell types that communicate to maintain homeostasis [18]. While architecture of normal tissue is lost in cancer, tumor cells maintain many interactions with surrounding non-malignant cells and extra-cellular matrix (ECM) to create a specific tumor contexture [19]. Both primary and metastatic lesions get infiltrated by diverse stromal cell types, including endothelial cells (ECs), immune cells, fibroblasts and bone marrow-derived cells such as macrophages, mast cells and mesenchymal stem cells (MSCs) [20]. Stromal cells contribute to cancer growth and metastasis, through modulation of different pathways [21-27]. The interactions between cancer and stromal cells are thus primordial for tumor biology as the corresponding crosstalk induces phenotype changes resulting in stromal “activation” and tumor promotion in both primary and metastatic sites [28,29]. Therefore, the microenvironment plays a major role in cancer spread, beyond tumor cell autonomous mechanisms [30] and might have clinical consequences [31].

Understanding the mechanisms governing the relationship between cancer and stromal cells is thus essential to target tumor progression. Among the different peritoneal cells, MSCs are a cornerstone for cancer spread through their participation in the establishment of the pre-metastatic niche and the induction of metastatic and chemo-resistant phenotypes [21,22,25,32-34]. Here we review the complex role of mesenchymal cells in ovarian cancer progression and subsequently illustrate the constitution of a multi-parameter “mesencrine” niche.

### Mesenchymal stem cells: a multipotent partner

**Figure 1:**
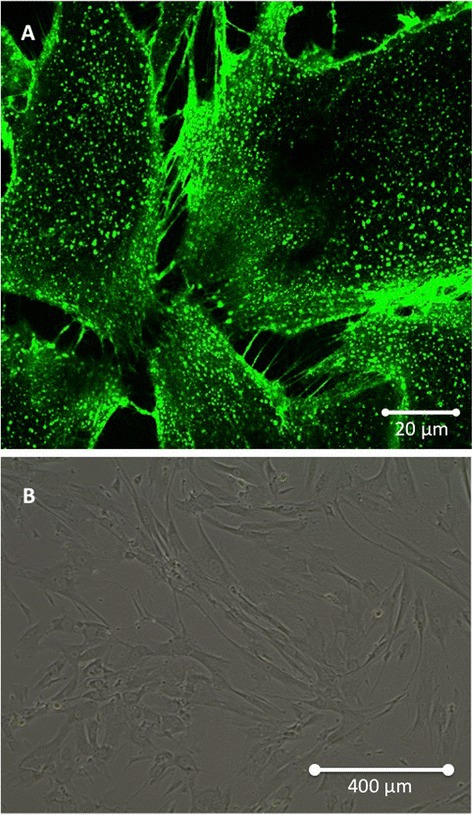
**Morphological aspect of Mesenchymal Stem Cells (MSCs) cultivated in vitro. (A)** Confocal microscopy showing intercellular interaction through Tunneling Nanotubes. **(B)** Optical microscopy illustrating the classical fibroblast-like shape of MSCs (×10).

Although they were initially described in the bone marrow [35-38], MSCs may participate to the constitution of the blood vessel walls and thus be ubiquitous cells, belonging to virtually all organs [39-41]. As a subset of pericytes, they can potentially originate from a perivascular niche [42-46]. MSCs are adherent cells that have a fibroblastic morphology (Figure 1). They are capable of forming colonies (termed colony-forming unit fibroblastic) when selected by adhering to plastic surfaces [38]. Their identification is based on diverse surface markers, including CD105, CD73, CD90, CD166, CD44 and CD29.

MSCs play a major role in tissue homeostasis through the following characteristics: stemness, self-renewal and multipotence [47]. Their participation in tissue repair has been suggested by their capacity to differentiate in different cell types including osteoblasts, adipocytes and chondrocytes [38,48-50]. However, they usually do not differentiate into resident cell types of injured tissue as supported by the therapeutic value of their conditioned media [51]. Furthermore they exhibit low and transient engraftment in the damaged organs [52]. Their reparative function may thus operate through paracrine (“mesencrine”) factors [53].

MSCs also serve as niche cells for other cell types, by regulating regenerative processes. Indeed, they participate to both hematopoietic cells expansion and hematopoietic stem cells (HSCs) regulation [54-57]. In particular, they contribute to self-renewal and differentiation of HSCs through their regulation of the osteogenic niche [58-61].

**Figure 2:**
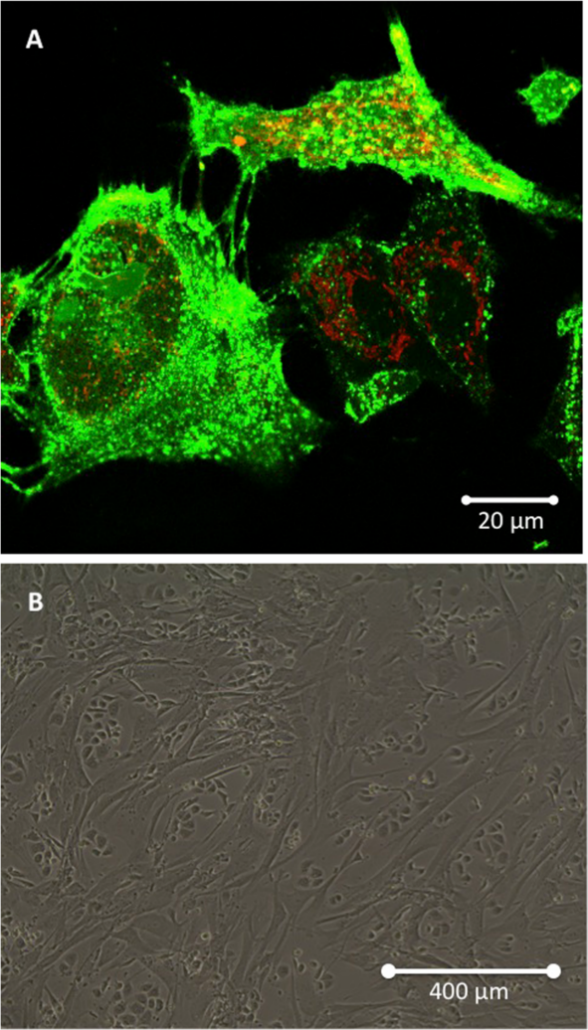
**Mesenchymal Stem Cells (MSCs) co-cultivated in vitro with Ovarian Cancer Cells (OCCs). (A)** Confocal microscopy: eGFP-MSCs (green) interact with PKH26-OCCs (red). **(B)** Optical microscopy showing how cancer and mesenchymal cells organize in vitro (×10).

MSCs have a primordial role in immune tolerance [42,62]. Their immunosuppressive effect on T lymphocytes and dendritic cells may prevent self-responses in both physiological and pathological conditions [63,64]. In addition to their regenerative and immunomodulatory properties, MSCs contribute to tissue healing through many trophic abilities, such as: (i) inhibition of apoptosis and fibrosis; (ii) stimulation of angiogenesis; (iii) recruitment and regulation (proliferation and differentiation) of stem and progenitor cells; (iv) attenuation of oxidative stress [65]. Therefore, they display a wide range of functions, including cell regeneration, immunomodulation and stimulation of angiogenesis. Considering their diverse abilities, MSCs may thus constitute a key cell in the neoplastic niche, as supported by their incorporation into the stroma of solid tumors [66-68] (Figure 2).

### MSCs engraftment into tumoral stroma leads to pro-tumoral crosstalk

#### Recruitment of cancer-associated MSCs

Bone marrow-derived MSCs are mobilized in blood circulation of patients presenting with advanced-stage ovarian cancer [34]. Interestingly, they normalize after complete cyto-reductive surgery. Such observation indicates a potential role for the interactions between ovarian cancer cells (OCCs) and MSCs during tumorigenesis.

Indeed, tumors act as unhealed wounds producing a continuous source of inflammatory mediators [69], resulting in the recruitment of other cell types, including MSCs [70-72]. Cancer cells hijack the cytokine machinery to acquire phenotypic advantages in proliferation [73], angiogenesis [74] and invasive and migratory properties [75-77]. The cytokine machinery is thus widely deregulated in advanced ovarian cancer, as supported by the amplification of many genes encoding cytokines in OCCs [17,78]. Such deregulation leads to bone marrow-derived and resident-tissue MSCs engraftment into tumor stroma through increased release of chemo-attractant soluble factors [79]. Many cytokines are involved in their recruitment, including IL-6, SDF1 (stroma derived factor 1), prostaglandine E2 (PGE2), PDGF and LL-37 (leucine, leucine-37) [21,80].

#### Educating MSCs to build a permissive tumoral environment

**Figure 3:**
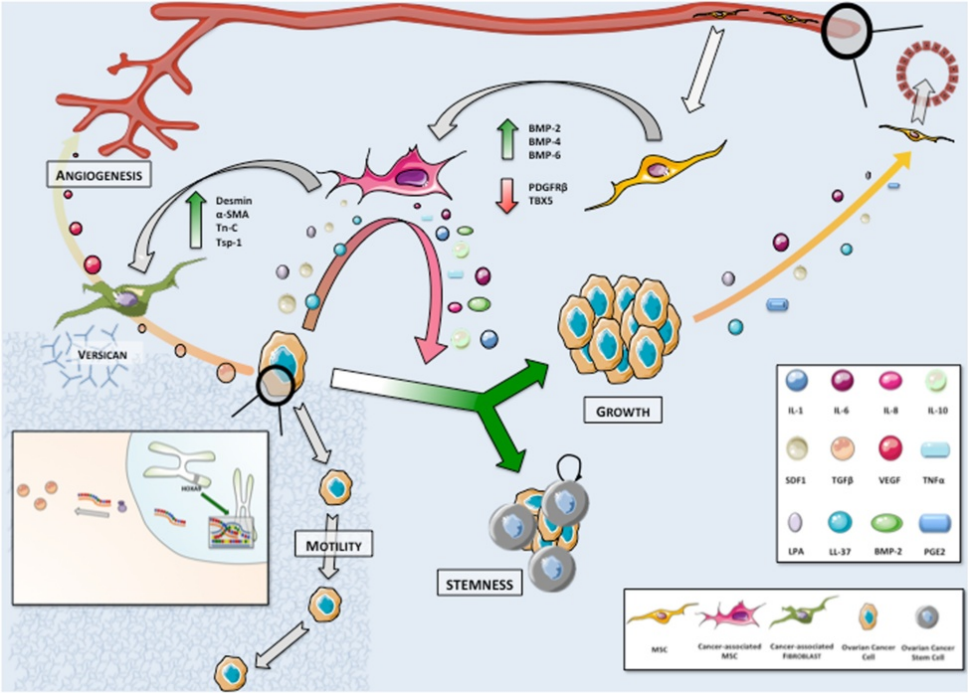
**The cytokine-mediated crosstalk between Mesenchymal Stem Cells (MSCs) and Ovarian Cancer Cells (OCCs) leads to a shift in MSCs phenotype resulting in tumorigenesis promotion.** MSCs are recruited to tumor stroma from a peri-vascular niche. Their engraftment is associated with phenotypic modulations in response to tumor-derived secreted factors. Primed MSCs support tumor growth and self-renewal. Their differentiation in cancer-associated fibroblasts (CAFs) contributes to stromal modifications suitable for tumor expansion and stimulates angiogenesis. We highlight the role of HOXA9 expression that results in transcriptional activation of the gene encoding TGFβ2 inducing in turn CAFs expression VEGF.

In ovarian cancer, the crosstalk between tumor and stromal cells leads to bilateral phenotype modulation. Indeed, besides OCCs phenotypic modifications, the phenotype of cancer-associated MSCs will evolve. Although it may differ from a cancer type to another, the shift in MSCs phenotype after their integration into tumor stroma mostly results in tumorigenesis promotion (Figure 3). For instance, breast cancer cells induce MSCs de novo CCL5 (RANTES) secretion which then acts as a paracrine mediator of increased motility, invasion and metastatic abilities of the tumor cells [68]. Once they engraft in ovarian neoplastic microenvironment, cancer-associated MSCs display an expression profile distinct from bone marrow MSCs, with an increased expression of BMP-2, BMP-4 and BMP-6, and a significant downregulation of PDGFRβ and TBX5 [81]. This pro-tumoral shift in phenotype is mediated, at least partially, by tumor-derived secreted factors.

LL-37 enhances MSCs secretion of IL-1β, IL-6, IL-8, IL-10 and TNFα while diminishing the secretion of IL-12 [82], with a positive impact on tumor growth. MSCs exposed to LL-37 stimulate endothelial cell tubule formation *in vitro* suggesting concomitant increased production of pro-angiogenic molecules. They also migrate around endothelial structures and acquire a pericyte-like differentiation [82]. Lysophosphatidique acid (LPA) is a small bioactive phospholipid produced by OCCs that stimulates differentiation of MSCs in myofibroblast-like cells [83-85]. These activated fibroblasts, also termed cancer-associated fibroblasts (CAFs), are a cornerstone in the establishment of tumor environment. MSCs incorporation into tumor stroma is thus associated with a morphological shift toward CAF-like phenotype, including expression of myofibroblast-like cell markers (α-SMA, desmin, VEGF), proteins involved in the regulation of ECM structure (Tn-C, Tsp-1, SL-1) and tumor promoting factors [22]. The underlying mechanism governing this differentiation process may also involve exosomes secreted by the tumor [86]. Interestingly, exosomes from different ovarian cancer cell lines (OVCAR-3 and SKOV-3) activate different MSCs signaling pathways (SMAD and AKT, respectively), suggesting that exosome content may vary according to cancer cell phenotype and thus modulate the tumor stroma differently. A genomic approach also correlates OCCs ability to induce CAFs features in MSCs with the expression of HOXA9, a Mullerian-patterning gene [87]. HOXA9 expression results in transcriptional activation of the gene encoding TGFβ2 that induces MSCs expression of IL-6, VEGF-A and SDF1. Schauer et al. have described a circuit whereby OCCs secrete IL-1β instructing a CAFs niche through p53 inhibition [31]. In return, the CAFs niche secretes IL-8, growth regulated oncogene-alpha (GRO-α), IL-6 and VEGF. Therefore, the modulation of MSCs phenotype contributes to generate a cytokine mediated inflammatory contexture suitable for tumor progression.

Once MSCs differentiate into CAFs they participate in the formation of fibrovascular networks within the tumor [22,88]. CAFs contribute to the perivascular matrix through the production of desmin and α-SMA [22]. CAFs secrete versican, a large ECM proteoglycan which production is up regulated by TGFβ via TGFβ-RII and SMAD signaling [89]. Up regulated versican then promotes OCCs motility. Their expression of the metalloprotease MMP-3 also participates in ECM regulation [22]. The resulting stromal modifications (increased vessel stability and matrix degradation) are compatible with tumor expansion, stimulated simultaneously by CAFs release of tumor-supportive growth factors, including HGF, EGF, IL-6 and SDF1 [88]. Ovarian tumors display increased expression of SDF1 in both CAFs and OCCs. SDF1 actively participates in the development of tumor environment and promotes tumor growth through complex mechanisms. First, it reduces local immunity and protects cancer cells by increased recruitment of plasmacytoid dendritic cell precursors resulting in poor anti-tumoral T cell activation through local overexpression of IL-10 and TNFα [90,91]. SDF1 also induces a dose-dependent proliferation of OCCs by its specific interaction with the receptor CXCR4, leading to transactivation of EGFR [92]. It participates as well in adhesion and trans-endothelial migration of cancer cells through MAP and Akt kinase regulation [93,94]. SDF1 promotes angiogenesis at tumor sites: hypoxia synchronously stimulates tumor SDF1 and VEGF production resulting in synergistic induction of angiogenesis [95]. SDF1 also acts as a chemo attractant for endothelial progenitor cells (EPCs) CXCR4 + [96].

Noteworthy, direct intercellular interaction participates in phenotypic and environmental changes. Indeed, we have shown in a co-culture setting that MSCs triggered pro-metastatic properties in OCCs, including adherence, invasion and migration through the modification of cancer cells transcriptomic profile [32].

#### Induction of tumor plasticity: the cancer stem cell (CSC) theory

The CSC theory if clinically confirmed may represent be an extreme form of cancer cell phenotypic plasticity. CSCs are defined with the following criteria: (i) self renewal, (ii) reproducible tumor phenotype, (iii) restricted to a minority among entire cell population, (iv) differentiation into non-tumorigenic cells, (v) expression of distinct cell markers allowing their isolation [97-100]. CSCs have been identified in many solid cancers, including ovarian malignancies [101-109]. The identification of ovarian CSCs is based on CD117 (c-kit), CD44, CD133 and ALDH markers as well as PKH67/PKH26 dyes [99,102,110-112]. According to Silva et al., dual positivity of CD133 and ALDH defines an effective cell population of ovarian CSCs [111]. In limited dilution assays, a small number of these cells are sufficient to initiate tumors. Furthermore, they exhibit an increased angiogenic ability compared to regular OCCs. In the clinical setting, the presence of CSCs in ovarian tumors portends poor survival outcomes, leading to increased tumor burden and chemoresistance [113,114]. Their persistence within a residual niche may therefore contribute to disease recurrences.

We have recently reviewed the role of the microenvironment in ovarian CSCs maintenance [115]. Among the diverse stromal actors, cancer-associated MSCs are determinant for the regulation of CSCs self-renewal via increased expression of BMP-2 [81]. MacLean et al. reported a 4- to 8-fold increase in the percentage of ovarian CSCs in the presence of cancer-associated MSCs. Interestingly, tumor stemness was only partially blocked by the BMP inhibitor Noggin, suggesting the existence of other redundant pathways. MSCs-derived IL-6 and IL-8, whose production is stimulated by LPA, participate in CSCs promotion: IL-6 contributes to self-renewal of CSCs and IL-8 to CSCs proliferation through its binding to CXCR1 receptor [72,102,103,116,117].

Altogether MSCs engraftment at primary tumor site positively impacts cancer progression, due to the combination of several mechanisms including two-sided phenotypic modulation and promotion of OCCs proliferation and angiogenesis. MSCs also contribute to build a suitable environment that will participate in the regulation of ovarian cancer metastasis.

### Metastatic niche: the concept of targeted spread

#### The “seed and soil” theory revisited

Based on autopsies of patients with breast cancer, Stephen Paget’s “seed and soil” theory illustrates the striking fact that a given tumor type will preferentially metastasize to specific organs [118]. This tumor tropism is clearly observed for certain cancers such as breast and prostate adenocarcinomas that commonly metastasize to bone tissue, or ovarian malignancies that typically spread into the peritoneal cavity. One century later, David Tarin reached a similar conclusion in patients with peritoneal carcinomatosis from diverse cancers including primary ovarian tumors [119]. While patients with peritoneo-venous shunts had millions of cancer cells poured in their blood circulation, they did not display more distant metastasis nor decreased survival compared to patients without shunts. Moreover, half of them did not develop any distant metastasis up to 27 months survival. Somehow, ovarian cancer is thus “programmed” to spread into selected organs and steer away from others. Besides the intrinsic abilities of cancer cells, tumor environment as well as resident stroma cells of distant sites participate in this targeted metastatic process.

#### The pre-metastatic niche

Lyden’s group has defined the concept of pre-metastatic niche as the early changes that occur in the future metastatic site before engraftment of cancer cells [79,120]. The constitution of such a niche dictates the pattern of metastatic spread. In brief, bone marrow-derived hematopoietic progenitor cells (HPCs) migrate to distant sites in response to growth factors and inflammatory cytokines secreted by the primary tumor. Kaplan et al. describe the existence of a complex loop where HPCs VEGFR1+ form cellular clusters in tumor-specific pre-metastatic sites before the arrival of cancer cells. Resident fibroblasts, possibly derived from MSCs and activated by tumor-specific growth factors, secrete fibronectin, an adhesion protein inducing VLA-4 (integrin α_4_β_1_) mediated recruitment of HPCs [29]. α_4_β_1_ signaling induces modifications of the local ECM mediated by MMP-9. The microenvironment alteration enhances recruitment of HPCs VEGFR1+, constituting in return a pre-metastatic contexture through a cytokine network including TNFα, MMP-9, TGFβ, and SDF1 [121]. SDF1 finally acts as a chemo-attractant for both hematopoietic progenitor cells (HPCs CXCR4+) and metastatic tumor cells (MTCs). In the hypoxia context, lysyl oxidase secreted by hypoxic tumor cells accumulates at pre-metastatic sites, resulting in CD11b + myeloid cell recruitment and increased production of MMP-2 [122]. MMP-2 favors tumor cells attachment through the changes they mediate in ECM [123]. MTCs subsequently engraft the permissive niche and contribute to the constitution of micro-metastases.

However, the stromal role during the metastatic process goes beyond the constitution of the pre-metastatic niche. Primary tumor stroma may also participate in selecting clones primed for metastasis in specific organs. In a triple negative breast cancer model, Zhang et al. have demonstrated that a tumor stroma rich in CAFs selects for cancer clones that fit to thrive on the CAF-derived cytokines SDF1 and IGF1 and sheds the carcinoma population toward a preponderance of such clones [124]. These clones display a constitutively high level of Src activity and bone metastatic ability contrary to most triple negative cancers that prominently metastasize to visceral organs. CAFs provide a cytokine contexture (SDF1 and IGF1) similar to bone marrow microenvironment and select metastatic seeds compatible with the target organ.

Altogether, data in the literature support the concept that the stroma actively participates in tumor promotion and to the constitution of a permissive niche for metastatic cells. It is thus a strong determinant of metastatic tropism. While different tumor types have their own physiopathology governing preferential sites for metastasis, ovarian cancer spread at an advanced stage (above IIIC) is limited to the peritoneum and distant blood-borne metastases are quite rare [125,126]. The recurrences are also often located within the abdominal cavity proposing the role of a residual niche within the peritoneum.

### Initial dissemination within peritoneal cavity

**Figure 4:**
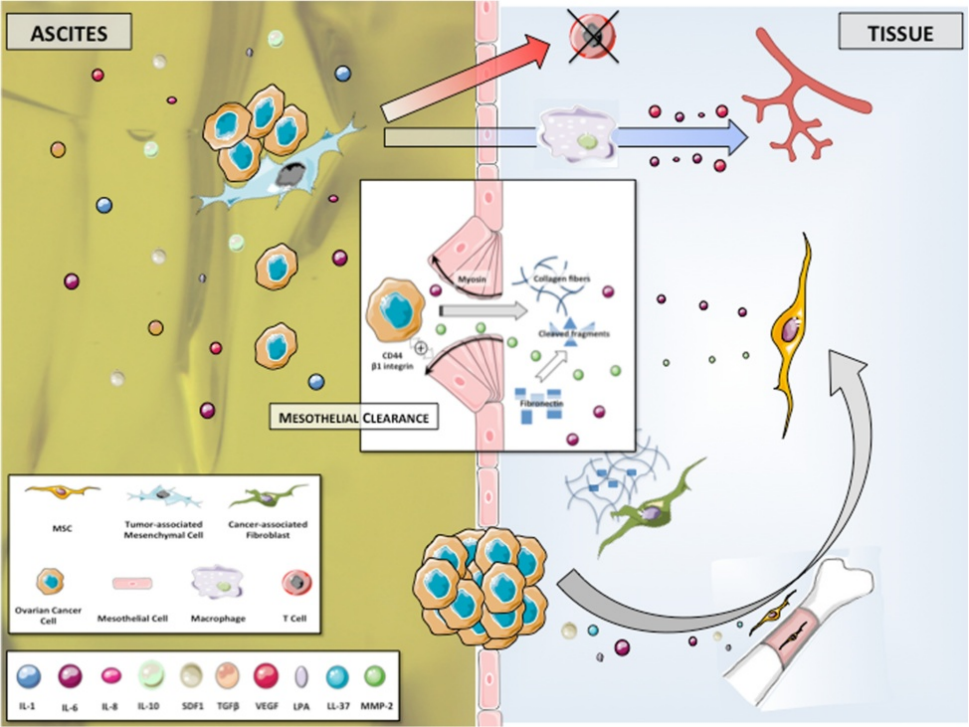
**Early steps in peritoneal infiltration.** Tumor-associated Mesenchymal Cells (TAMCs) constitute a protective niche for ascitic Ovarian Cancer Cells (OCCs) through their inhibition of anti-cancer T cells. They also participate in angiogenesis at metastatic sites by inducing macrophages production of pro-angiogenic cytokines including IL-6, IL-8 and VEGF. Ascitic OCCs may be the primary source of peritoneal metastases. The initiation of peritoneal invasion relies on the ability of OCCs to attach to and to clear the mesothelial cells that constitute the peritoneum. This mesothelial clearance involves integrin- and talin-dependent activation of myosin and allows OCCs to get access to the basement membrane. Bone marrow-derived Mesenchymal Stem Cells (MSCs) are recruited at metastatic sites and favor the infiltration process with the release of secreted factors such as IL-6 and MMP-2. The metalloprotease MMP-2 is also up regulated in OCCs upon binding to mesothelium and leads to improved attachment of tumor cells by modifying the extracellular matrix.

Initial dissemination of OCCs from primary tumor is based on changes in expression of cellular adhesive proteins (Figure 4). Intercellular adhesion in ovarian tumors is mediated by N- and E-cadherin, and cell-matrix adhesion by integrins [127]. Disruption of both cell-cell contact and cell-matrix interactions results in the shedding of single cells or muticellular aggregates into peritoneal cavity. These ascitic OCCs undergo epithelial-to-mesenchymal-transition, resulting in a phenotype with low levels of E-cadherin and higher invasiveness and motility compared to primary tumor cells [128]. They also display CSCs characteristics when clustered in compact spheres, leading to increased chemoresistance and tumorigenesis [99]. They migrate to distant areas following the flow of peritoneal fluid hence the geographical localization of lesions within the abdomen at diagnosis. Ascites fluid comprises more than 200 proteins in its soluble fraction, including LPA, SDF1, cytokines such as RANTES, IL-1, IL-6, IL-8 and IL-10, growth factors (EGF, VEGF, HB-EGF, TGFβ, TNFα and CSF1) and ECM proteins such as collagens I and III [129-136]. It thus constitutes a suitable environment for OCCs survival.

Cellular components including inflammatory and mesothelial cells are also prevalent in ascites. We have isolated stromal cells from ascites of patients presenting with ovarian cancer [137]. These tumor associated mesenchymal cells (TAMCs) were closely associated with tumor cells (photo) and shared some homology with bone marrow- and adipose- derived MSCs (CD9, CD10, CD29, CD146, CD166, HLA-1) [24]. They might therefore represent a differentiated stromal subset of MSCs. Converging data support that TAMCs actively contribute to metastatic process through different mechanisms. We have shown that intra-peritoneal co-injection of TAMCs and OCCs conferred a proliferative advantage to OCCs in a murine model with enhanced tumor growth and development of neoplastic ascites [24]. Noteworthy, TAMCs were preferentially localised close to cancer cells, typically in the periphery of tumors. Moreover, the presence of TAMCs was associated with increased tumor vascularization. Concordantly, HIF-1α and VEGF-A were overexpressed in tumors and ascites, respectively, derived from the group that received co-injection. Castells et al. failed to demonstrate any proliferative effect of TAMCs on ECs [138]. Nevertheless they have shown the existence of a crosstalk between TAMCs and macrophages yielding increased production of pro-angiogenic cytokines, including IL-6, IL-8 and VEGF.

TAMCs also display a protective role by inducing chemoresistance and immunomodulation [23,137,139]. TAMCs inhibit both proliferation and cytokine production in human CD4 and CD8 T-cells, allowing cancer cells to evade immune surveillance. Therefore, the protection TAMCs provide prompts us to consider them as a niche where tumor cells are sheltered against therapy.

Cancer dissemination in ovarian malignancies may be sequential, from the primary tumor to abdominal “milieu” and then to the peritoneum. Indeed, ascitic OCCs might be the primary source of intraperitoneal metastatic lesions. Their attachment to the peritoneum constitutes the first step toward peritoneal infiltration.

### Peritoneal carcinomatosis

#### Peritoneal involvement

**Figure 5:**
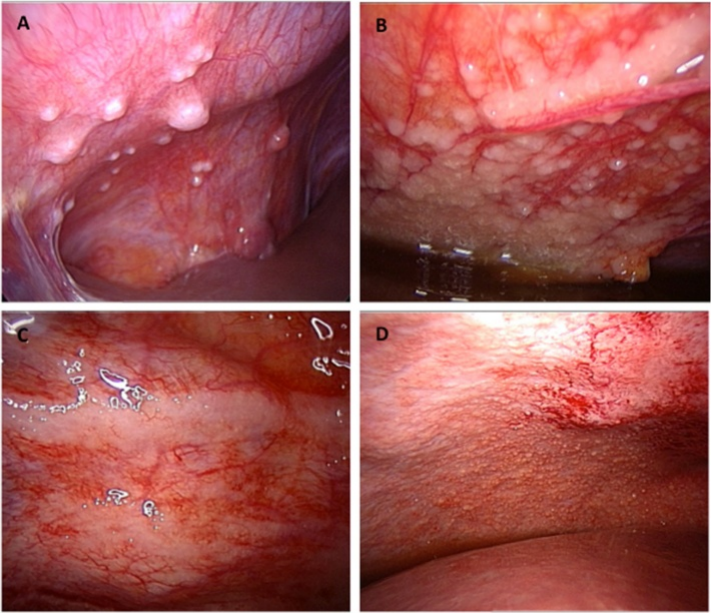
**Macroscopic aspects of peritoneal carcinomatosis. (A)** Isolated lesions. **(B)** Confluent lesions. **(C)** Typical “taches de bougie” lesions. **(D)** Miliary lesions.

**Figure 6:**
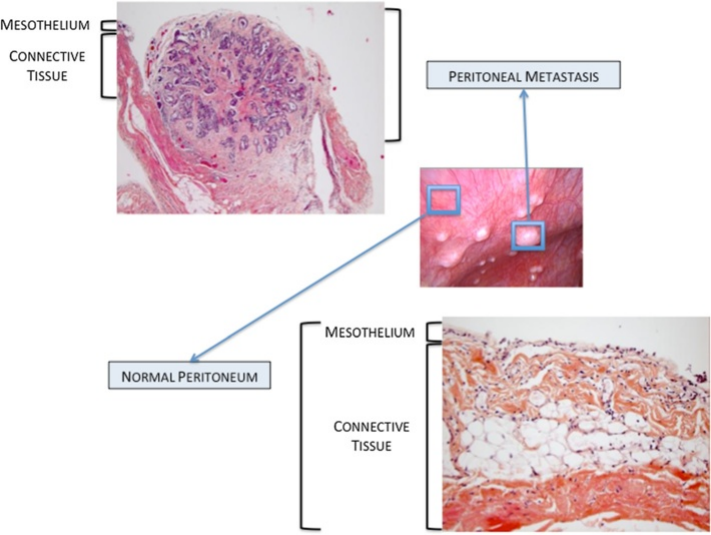
**Pathological aspects of normal peritoneum and peritoneal metastasis.**

Clinical presentation at baseline in patients with advanced stages includes ascites, peritoneal carcinomatosis and omental involvement [140] (Figure 4). The peritoneum is a complex organ constituting the microenvironment of ovarian cancer metastatic nodules (Figure 5). It is composed of a continuous mesothelial cell layer covering all abdominal organs except ovaries. The peritoneum lies on a basement membrane covering stromal tissue. This stroma contains a collagen-based matrix (collagen types I and III), blood and lymphatic vessels, nerve fibers and fibroblastic-like cells (Figure 6). The peritoneum is considered as a tertiary lymphoid organ that allows fast mobilization and recruitment of the inflammatory machinery to overcome abdominal injury or infection. Hence many inflammatory cytokines are up regulated in ovarian cancer ascites. Mesothelial cells also contribute to peritoneal fluid dialysis, abdominal healing and formation of adherences [141,142]. Therefore, the peritoneum constitutes a functional and anatomical barrier against intra-abdominal aggression and is considered as the first line of defense against cancer spread [143,144].

However the metastatic process manages to disrupt the organisation of the mesothelial layer: during the formation of carcinomatosis nodules, mesothelial cells aggregate around the neoplastic lesion [145]. The mechanism used by OCCs to clear mesothelial cells and get access to the basement membrane is complex and yet poorly understood. Tumoral soluble factors may prime mesothelial tissue for cancer spread. Indeed, mesothelium in ovarian carcinomatosis displays morphological changes and forms a discontinuous layer of hemispheric cells [146]. This phenotypic alteration is associated with modifications in transcriptomic profile, including modulation of genes involved in inflammation, catalytic activity, cellular adhesion and ECM constitution [147]. Analysis of surgical specimens has suggested that mesothelial cells may nurture peritoneal metastases through the production of growth factors such as VEGF and fibroblast growth factor 2 (FGF-2) [148]. The initiation of peritoneal invasion also relies on the ability of OCCs to attach to mesothelial cells through activation of CD44 and beta-1 integrin [149,150]. In their *in vitro* model, Iwanicki et al. have shown that ovarian cancer spheroids use integrin- and talin-dependent activation of myosin and traction force to promote displacement of mesothelial cells [151]. This mesothelial clearance permits OCCs to get access to the basement membrane and to stromal cells that will then support their survival and growth. MSCs play an important role along this process, as supported by our 3D model of early peritoneal infiltration based on amniochorionic membrane [33]. In serum free condition, OCCs became adherent to the membrane within the first 24 hours following incubation and started to infiltrate the stroma 48 hours after adhesion. Infiltration was significantly deeper in areas settled with MSCs, due to increased release of IL-6. Therefore, MSCs generate a cytokine contexture suitable for metastasis establishment. Metalloproteases such as MMP-2 contribute through a feed-forward loop to cancer cells peritoneal adhesion and invasion. MMP-2 expression is up regulated in OCCs upon binding to mesothelium, through direct cell-cell interaction involving mesothelial cells [123]. MMP-2 is also produced by bone marrow-derived MSCs recruited to the tumor site in response to OCCs secretion of LL-37 [21]. MMP-2 over-expression leads to increased degradation of ECM proteins such as vibronectin and fibronectin. OCCs adhere more efficiently to cleaved fragments, resulting in improved attachment. Noteworthy, vibronectin and fibronectin production is increased during the metastatic process. Resident fibroblasts, potentially deriving from resident MSCs, are primed by tumor specific growth factors and and constitute the main source of ECM proteins [29].

#### Omental infiltration

**Figure 7:**
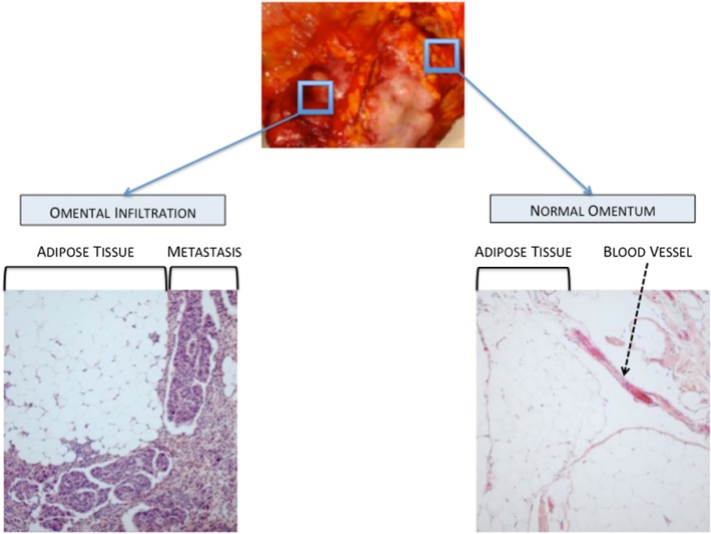
**Pathological aspects of normal omentum and omental metastasis.**

The omentum is a large fold of visceral peritoneum containing fatty tissue. In a 3D culture model of omental infiltration, Kenny et al. have demonstrated that OCCs preferentially adhere to and invade through collagen I and IV rather than fibronectin, vitronectin and laminin [143]. Furthermore, resident cells differently impact the metastatic process. While mesothelial cells inhibit both adhesion and invasion, omental fibroblasts promote OCCs attachment and infiltration. Similarly to peritoneal infiltration, MMP-2 over-expression observed upon the interaction between OCCs and omental fibroblasts may promote tumoral infiltration [123]. Omental MSCs (O-ASCs) also participate in the invasion process [152]. In a model of endometrial carcinoma, O-ASCs stimulated cancer cells proliferation and promoted *in vivo* tumor growth and vascularization [152]. Compared to the control group, the tumors associated with O-ASCs contained a more mature and extensive fibrovascular network. These findings can be extrapolated to the omental invasion occurring in ovarian cancer (Figure 7).

### MSCs and chemoresistance

#### Microenvironement mediates resistance to therapy

The mainstay of treatment for ovarian cancer involves complete cyto-reductive surgery associated with platinium and taxane-based chemotherapy [153]. However, most patients achieving complete initial clinical remission will develop recurrences and resistance to first line drugs [154-156]. Acquired chemoresistance involves many mechanisms: (i) alteration of the lipid membrane modifying drug penetrance; (ii) increased capacity in DNA repair; (iii) modification of drug targets; (iv) drug inactivation mediated by metallothionein- or glutathione-dependent mechanisms; (v) loss of drug surface transporter; (vi) drug clearance by efflux pump [137]. Such resistance mechanisms consist in long-term processes and usually arise after multiple courses of chemotherapy [155,157]. They develop over time as a result of sequential genetic changes that ultimately culminate in some complex therapy-resistant phenotypes.

However, acquired chemoresistance cannot explain most of treatment failures in ovarian cancer. The majority of recurrences will occur within the first two years following completion of initial treatment. Primary resistance to treatment only concerns a low subgroup of early relapses (up to 6 months after initial treatment). Other diseases are considered platinium-sensitive. Therefore, in addition to casual mechanisms of acquired resistance, the microenvironment might mediate a kind of *de novo* drug resistance leading to the persistence of a hidden residual disease responsible for a subsequent relapse.

First evidences were provided in 1990 by Teicher et al. [158]. In a murine breast cancer model, long-term exposure to treatment was responsible for chemoresistance in tumor-bearing animals. Nevertheless, in spite of high levels of *in vivo* resistance, no significant resistance was observed when cancer cells were exposed to the same drugs *in vitro*. These findings set up the stage for the paradigm that drug resistance can develop through mechanisms that are expressed only *in vivo* and may involve the crosstalk between cancer and stromal cells. Somehow the microenvironment could protect the tumor cells from the effects of anticancer agents.

The cross talk between cancer and stromal cells is responsible for changes in ECM and cytokines release. Reciprocal integrin- and soluble factor-mediated interaction induces in cancer cells a transient drug resistant phenotype leading to persistence of surviving cells [157]. Over time, genetic instability inherent to tumor cells combined with selective pressure of therapy will result in successive and random genetic changes. Such modifications will cause the gradual development of more complex and permanent acquired-resistance phenotypes, and relapses may originate from these persistent cancer cells.

#### MSCs actively contribute to environment-mediated resistance

The microenvironment provides a transient protection while OCCs are acquiring genetic changes. Oncologic trogocytosis perfectly illustrates this mechanism: through a membrane uptake, cancer cells can get new functionalities. We have observed such hetero-cellular interaction between TAMCs and OCCs [137]. Trogocytosis allowed OCCs to acquire functional multidrug resistance proteins from TAMCs membrane, including P-gp and LRP. Such mechanisms have been described in other tumors as well [159,160].

MSCs are responsible for phenotypic modulation toward more aggressive cancer cell clones. We have proposed a transcriptomic approach in OCCs co-cultured with MSCs [32]. The analysis revealed that 3 biological-function gene clusters were enriched in OCCs upon contact with MSCs, comprising metastatic, proliferative and chemoresistance abilities. Concordantly, OCCs co-cultured with MSCs displayed chemoresistance to taxol and carboplatin. MSCs-derived secreted factors are also able to confer chemoresistance to platinium in OCCs, as supported by the decrease of carboplatin-induced apoptosis in the presence of MSCs condition medium [139]. This apoptosis blockade is mediated by the activation of the phosphatidylinositol 3-kinase/Akt signaling pathway and the phosphorylation of its downstream regulator X-linked inhibitor of apoptosis (XIAP).

Microenvironment protection may have contributed to the disappointing results of hyperthermic intraoperative chemotherapy (HIPEC) for stage IIIC ovarian cancers [161,162]. HIPEC procedure involves a complete resection of abdominal disease followed by intra peritoneal infusion of Oxaliplatin at 42-44°C. We have demonstrated that hyperthermia did not challenge survival of bone marrow-derived and cancer-associated MSCs [25]. Furthermore, in the context of hyperthermia, MSCs induced a thermo tolerance in OCCs through SDF1 secretion. The inhibition of SDF1/CXCR4 interaction restored cytotoxicity of hyperthermia.

Circulating MSCs, observed in advanced-stage ovarian malignancies, are also involved in chemoresistance. They are activated by platinium-derived drugs and in turn secrete fatty acids (PIFAs) that, in discrete quantities, confer OCCs resistance to several types of anti-cancer agents [34]. PIFAs induce acute and reversible prevention of apoptosis in cancer cells through an indirect and yet undetermined effect that can be prevented by concomitant infusion of cyclooxygenase-1 inhibitor.

MSCs widely participate in drug resistance in ovarian cancer through many mechanisms. Anti-tumor therapy should thus be associated to additional therapies targeting tumor-stroma interactions. Such therapeutic combination would prevent from temporary microenvironment-mediated drug resistance and might suppress any minimal residual disease. In the era of personalized medicine, it represents one of the biggest challenges for further therapeutic approaches in ovarian cancer.

## Conclusion

**Figure 8:**
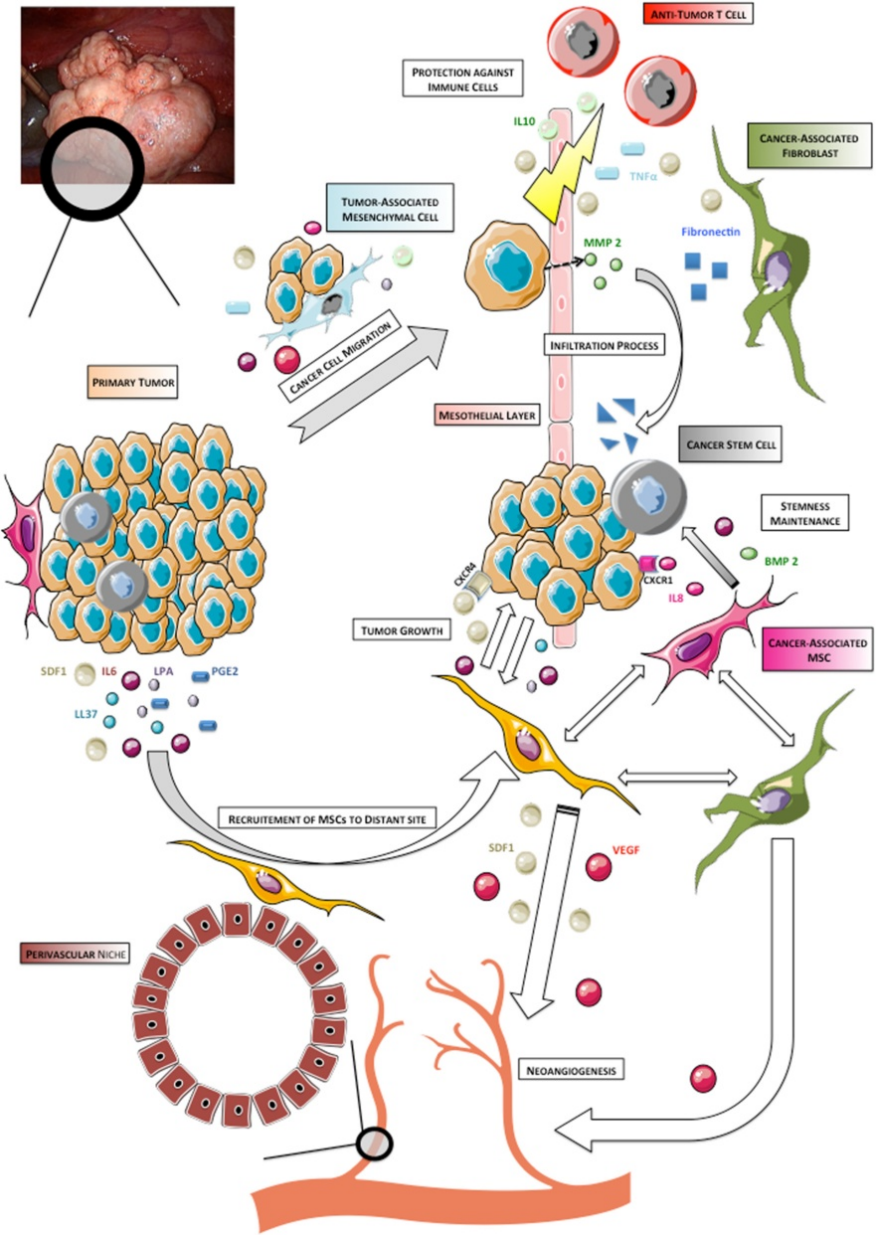
**Overview of the role of Mesenchymal Stem Cells along tumorigenesis.**

Tumor stroma and microenvironment represent a cornerstone in the regulation of OCCs behavior (Figure 8). MSCs contribute to each step of cancer spread, from proliferation to chemoresistance, from infiltration to metastasis. The crosstalk between MSCs and OCCs is based on complex mechanisms, involving cell-cell interaction and secreted factors. Beyond casual phenotypic changes they generate in tumor cells, MSCs provide a smart environment-mediated resistance protecting residual disease from treatment while acquired mechanisms are developing. OCCs and MSCs clearly constitute a deadly cocktail, offering the disease a multi-potent partner. Therefore, to enhance suppression of any residual disease, future therapeutic approaches should thus combine anti-tumor therapy to new molecules targeting tumor-stroma interactions.

## Abbreviations

ECM: Extra-cellulra matrix
EC: Endothelial cell
MSC: Mesenchymal stem cell
HSC: Hematopoietic stem cell
OCC: Ovarian cancer cell
CAF: Cancer associated fibroblast
EPC: Endothelial progenitor cell
CSC: Cancer stem cell
HPC: Hematopoietic progenitor cell
MTC: Metastatic tumor cell
TAMC: Tumor associated mesenchymal cell
HIPEC: Hyperthermic intraoperative chemotherapy
